# Workflow-dependent noise peaks and exposure assessment in operating rooms: implications for occupational risk

**DOI:** 10.1093/annweh/wxag051

**Published:** 2026-07-10

**Authors:** Hamdiye Banu Katran, Nermin Ocaktan, Ece Uzun, Gulcan Altinkaynak

**Affiliations:** Department of Nursing, Faculty of Health Sciences, Marmara University, Başıbüyük Yolu No:4/B 34854, Istanbul, Türkiye; Department of Nursing, Faculty of Health Sciences, Acibadem Mehmet Ali Aydinlar University, Içerenköy Kayışdaği cad. No:32 34638, Istanbul, Türkiye; Şişli Hamidiye Etfal Training and Research Hospital, Huzur mah. Cumhuriyet ve Demokrasi cad. 34453, Istanbul, Türkiye; Şişli Hamidiye Etfal Training and Research Hospital, Huzur mah. Cumhuriyet ve Demokrasi cad. 34453, Istanbul, Türkiye

**Keywords:** occupational noise exposure, task-based exposure measurement, exposure assessment, workflow-stage variability, workplace exposure, peak noise levels

## Abstract

**Background:**

Operating room noise is a recognized occupational exposure with potential implications for worker health and performance. Most workplace noise risk assessments rely on time-averaged levels, which may obscure short-duration, high-intensity exposure peaks occurring during specific workflow phases. Task-aligned exposure characterization may therefore provide additional value for occupational risk assessment.

**Methods:**

A field measurement study was conducted in 26 operating rooms under routine clinical conditions. A total of 611 real-time sound level measurements were obtained across 13 predefined surgical workflow stages using a calibrated sound level meter. Stage-specific noise levels were recorded to characterize exposure variability and peak events. Surgical nurses completed the Perceived Stress Scale and the Attention Control Scale immediately before and after procedures. Descriptive statistics, paired comparisons, and correlation analyses were performed.

**Results:**

The mean noise level across workflow stages was 66.57 dB, while short-term peak levels reached up to 87 dB. Peak exposures clustered in workflow stages involving equipment movement and patient transfer. Noise variability differed substantially between workflow stages. No statistically significant pre- to post-procedure differences were observed in perceived stress or attention control scores. Associations between stage-averaged noise levels and psychological measures were weak and not statistically significant.

**Conclusion:**

Reliance on average noise levels alone may underestimate task-dependent peak exposures in operating rooms. Workflow-aligned measurement approaches improve exposure characterization and may strengthen occupational noise risk assessment. Integrating stage-specific monitoring into routine workplace exposure evaluation may support more precise hazard identification and targeted risk control strategies for operating room personnel.

What’s important about this paper? This study presents field-based, workflow-aligned noise measurements in operating rooms, highlighting that short task-dependent peaks may not be fully captured by average-based assessments. The findings suggest that higher noise levels tend to occur during coordination-critical workflow transitions rather than remaining constant throughout procedures. This approach may help improve the identification of context-specific noise conditions and inform more targeted noise management strategies in clinical environments.

## Introduction

Operating rooms are among the most demanding clinical environments in healthcare. Surgical teams work under time pressure, manage complex technologies, and make rapid decisions that directly affect patient safety. In these settings, risks do not develop in a constant or predictable way but often emerge during short, workflow-dependent critical moments, particularly when multiple tasks and team interactions occur simultaneously (World Health Organization 1999; National Institute for Occupational Safety and Health 2018). When such variable risks are evaluated only through average exposure values, important hazards may remain unrecognized (Occupational Safety and Health Administration 2013).

Noise is a well-documented occupational hazard in operating rooms. Previous studies have shown that elevated noise levels can interfere with communication, distract team members, and negatively affect task performance and team coordination (Giv et al. 2017; Arabacı and Önler 2021; Peng et al. 2022). In clinical practice, excessive noise has been associated with communication failures during patient transfer, delayed responses to alarms, and increased susceptibility to error, all of which may compromise patient safety (Padmakumar et al. 2017; Keller et al. 2018; McLeod et al. 2021). Despite this evidence, operating room noise is still frequently perceived as an unavoidable background condition or a comfort issue rather than a workflow-related occupational risk factor (Giv et al. 2017; Fu et al. 2021).

Most research on operating room noise has relied on average sound levels measured over extended periods or limited procedural phases (van Pelt and Weinger 2017; Levinskas et al. 2019; Gülşen et al. 2021). Although such measurements provide general exposure estimates, they may not capture short-duration, high-intensity noise peaks. These peaks commonly occur during workflow transitions such as patient transfer, equipment movement, anesthesia induction, and team reorganization (Arabacı and Önler 2021; Nasri et al. 2023). From a safety perspective, these brief high-intensity periods may be more consequential than overall mean noise levels because they coincide with phases requiring uninterrupted communication and sustained attention (Ginsberg et al. 2013; Occupational Safety and Health Administration 2013).

Surgical nurses are continuously present across perioperative workflow stages and play a central role in maintaining communication, coordination, and continuity of care (Giv et al. 2017; Grant et al. 2021). Their responsibilities require sustained situational awareness, rapid task switching, and interprofessional collaboration. However, occupational noise exposure among surgical nurses is typically evaluated using global environmental thresholds that do not reflect task-specific or workflow-dependent variability (van Pelt and Weinger 2017; Gülşen et al. 2021). Furthermore, commonly used psychological indicators such as perceived stress and attention control may have limited sensitivity in detecting immediate or cumulative effects of short-term peak noise exposure, particularly among experienced staff who may demonstrate adaptive coping responses (Khademi and Khademi 2015; Kol et al. 2015).

International organizations, including the World Health Organization and the National Institute for Occupational Safety and Health, have established recommended noise exposure limits relevant to healthcare environments (World Health Organization 1999; National Institute for Occupational Safety and Health 2018). Occupational exposure limits for noise are commonly defined as 85 dBA over an 8-h time-weighted average for hearing conservation, while lower levels (approximately 60 to 70 dB) may already interfere with communication and cognitive performance (NIOSH 1998; WHO 1999; Basner et al. 2014). However, existing guidance provides limited methodological direction on how fluctuating, task-related exposure patterns should be incorporated into routine occupational risk assessments. Consequently, a gap remains between formal exposure standards and the dynamic exposure conditions encountered during real surgical workflow (Occupational Safety and Health Administration 2013; Fu et al. 2021).

Within an occupational hygiene framework, operating room noise should be regarded as a task-variable occupational exposure rather than a stable environmental characteristic. Exposure assessment strategies based solely on time-averaged measurements may therefore result in exposure misclassification and incomplete risk characterization. To address this limitation, the present study applies a workflow-aligned, stage-based measurement strategy to characterize real-time noise exposure across standardized phases of surgical care. Using a task-based exposure assessment approach, the study aims to improve occupational noise risk evaluation and support more precise hazard identification and exposure control planning in operating room environments.

It should be noted that the measurements in this study represent environmental noise levels at specific workflow stages rather than personal noise exposure. However, such stage-based measurements provide valuable insight into identifying high-risk moments within the surgical workflow and may support the prioritization of targeted control strategies.

## Materials and methods

### Research design

This study was designed as a descriptive correlational study with a pre–post measurement approach to assess stage-based environmental noise levels in the operating room and to examine the relationship between surgical nurses’ pre- and postoperative attention control and perceived stress levels. The study was conducted under routine clinical working conditions, without introducing any experimental interventions. No additional external noise sources were introduced, nor were any noise reduction measures implemented. This approach ensured that the recorded noise levels reflected typical workflow conditions experienced by nurses in the operating room.

### Research setting and time period

The study was carried out in 26 operating rooms of a training and research hospital in Istanbul between March and April 2024. The hospital included operating rooms for various surgical specialties, including general surgery, orthopedics, neurosurgery, gynecology, and urology. To reduce variability in workflow patterns and increase the comparability of measurements across operating rooms, data collection was conducted during the first surgical procedure of the day in each operating room. This strategy was intended to limit the influence of additional noise factors associated with increased workload, staff turnover, and consecutive surgeries throughout the day.

### Population and sample

The study population consisted of 55 nurses working in the operating rooms of the hospital. Forty-seven nurses who met the inclusion criteria and voluntarily agreed to participate were included in the sample. Inclusion criteria included active employment in the operating room for at least 6 mo, direct involvement in the first surgical procedure of the workday and voluntary participation. Exclusion criteria included being on medical or sick leave, not being on active duty on the day of data collection, reassignment to a different unit during the study period, and incomplete completion of pre- or postoperative scale forms.

Eight nurses were excluded due to medical or sick leave (*n* = 3), reassignment to another unit or operating room during the surgical workflow (*n* = 3), and incomplete scale forms (*n* = 2). Including a large proportion of the accessible population and achieving a sample size comparable to those reported in similar studies (Keller et al. 2018; Gülşen et al. 2021) supports the adequacy of the final sample.

### Sample size rationale

Because this study was designed as a descriptive and correlational investigation, a formal power analysis was not performed. Instead, sample sizes reported in similar studies (Keller et al. 2018; Gülşen et al. 2021; approximately *n* = 30 to 60) were considered, and a strategy of including all eligible nurses was adopted to ensure that noise measurements could be collected in each operating room. This approach increased the coverage of the accessible population and allowed comparison of noise profiles across different workflow conditions. Therefore, the dataset obtained from 47 participants was considered adequate for descriptive and correlational analyses.

### Data collection tools

Data were collected using the Nurse Identification Form, the Decibel Measurement Monitoring Form, the Perceived Stress Scale, and the Attention Control Scale. The researcher-developed forms were pilot-tested with 5 nurses prior to data collection to assess clarity and applicability. No revisions were required, and the forms were used in the main study without modification.

#### Nurse Identification Form

The Nurse Identification Form, developed by the researchers based on the literature, consisted of 8 items addressing age, gender, educational status, length of professional experience, length of operating room experience, surgical specialty, type of surgical procedure, and procedure date. This form was used to describe the sociodemographic and professional characteristics of the nurses and to control for individual factors that may be associated with noise exposure and psychological variables (Gülşen et al. 2021; Peng et al. 2022).

#### Decibel Measurement Monitoring Form

The Decibel Measurement Monitoring Form was developed by the researchers based on previous studies examining operating room noise (Ginsberg et al. 2013; Keller et al. 2018; Peng et al. 2022). The form included 13 standardized workflow stages expected to influence noise levels, including before patient admission, admission to the operating room, transfer from stretcher to operating table, anesthesia induction, positioning of the surgical team, first incision, entry of additional personnel, door opening, introduction of additional equipment, patient awakening, transfer from table to stretcher, patient removal from the room, and after the patient left the operating room.

The form was completed for each operating room by a trained researcher to ensure consistency in data collection and to minimize observer variability.

#### Perceived Stress Scale

The Perceived Stress Scale was developed by Cohen et al. (1983), and its original Cronbach's alpha coefficient was reported as 0.86 (Cohen et al. 1983). In this study, the 8-item Turkish adaptation by Bilge et al. was used, with a reported Cronbach's alpha of 0.81 (Bilge et al. 2009). The scale consisted of a 5-point Likert-type response format (0 = never, 4 = very often), with 3 reverse-scored items (items 4, 5, and 6) and 5 directly scored items (items 1, 2, 3, 7, and 8). Total scores ranged from 0 to 32. The scale included 2 subdimensions: perceived stress (items 1, 2, 3, 7, and 8) and perceived coping (items 4, 5, and 6). Higher total scores indicated higher levels of perceived stress. In the present study, internal consistency was high, with a Cronbach's alpha of 0.87.

#### Attention Control Scale

The validity and reliability study of the 1D, 20-item Attention Control Scale developed by Derryberry and Reed was conducted in Turkish by Akın et al. (2013). The scale contained no reverse-scored items and used a 4-point Likert-type response format (1 = almost never, 2 = sometimes, 3 = often, 4 = always). The Cronbach's alpha coefficient reported in the Turkish adaptation study was 0.88. Higher scores indicated higher levels of attentional control. In this study, internal consistency was high, with a Cronbach's alpha value of 0.89.

The Perceived Stress Scale and Attention Control Scale were selected due to their validated use in assessing psychological stress and attentional regulation in healthcare settings. Unlike workload-focused instruments such as the NASA Task Load Index, these tools were considered more appropriate for capturing subjective stress perception and attentional control in routine clinical environments.

### Timing of scale administration

All scales were administered at 2 time points. The preoperative assessment (pretest) was completed after the nurses arrived in the operating room at the beginning of the workday and immediately before the initiation of surgical preparation. The postoperative assessment (posttest) was conducted within 10 min after completion of the surgical procedure, before the nurse left the work area.

### Noise measurement method

Noise levels were measured using a calibrated, battery-operated digital sound level meter (UNI-T, Model UT353), with measurements recorded in A-weighted decibels (dB[A]) in accordance with standard occupational noise assessment practices. The device was calibrated by the institution's biomedical engineering department prior to data collection (15 July 2024; calibration No: 121), and daily field checks were performed following manufacturer guidelines. The same instrument was used for all measurements, and it met performance characteristics consistent with IEC Class 2 sound level meter specifications.

Noise measurements were conducted as stage-based environmental assessments across 13 predefined phases of the surgical workflow, including patient admission, anesthesia induction, surgical preparation, equipment introduction, patient awakening, and removal from the operating room. Measurements were recorded as instantaneous (FAST-response) point readings rather than continuous monitoring, with each stage assessed once per participating nurse. These measurements represent short-duration environmental sound level readings and should be interpreted as localized environmental measurements rather than time-integrated or full-shift personal exposure estimates. The minimum and maximum values reported represent the range of repeated instantaneous measurements across observations within each workflow stage and do not reflect time-integrated noise levels. Overall, 611 measurements were obtained from 47 nurses across the standardized workflow stages.

All measurements were performed by a single trained researcher to ensure consistency and minimize observer variability. The sound level meter was positioned approximately 1.5 m from the operating table and 1.5 m above the floor, corresponding to the typical working distance and standing height of surgical nurses, and was kept isolated from contact surfaces to avoid vibration interference.

### Measurement protocol, environmental recording, and bias control

Environmental conditions were recorded using a structured observation form by the same investigator during each measurement session, including the number of personnel present, surgical specialty, active equipment (eg, laparoscopic units, aspirators), frequency of door openings, room temperature, ventilation conditions, and background noise sources (eg, monitor alarms, ventilator sounds). These variables were documented to support contextual interpretation of the measured noise levels and to account for potential confounding factors.

To reduce measurement variability, all decibel measurements were performed by the same investigator, and device positioning, measurement timing, and recording procedures were standardized across operating rooms. Data collection was limited to the first surgical procedure of the day to reduce variability related to workflow differences across procedures. Any unusual circumstances (eg, emergency cases or unexpected team changes) were documented and considered during data interpretation.

### Ethical aspects of the research

Ethical approval for the study was obtained from the Ethics Committee of Şişli Hamidiye Etfal Training and Research Hospital (Decision No: 4332, dated 12 March 2024). Permission for data collection was granted by the hospital administration (Decision No: 2024/11, dated 10 July 2024). All participants were informed both verbally and in writing about the purpose and scope of the study, the voluntary nature of participation, and their right to withdraw at any time without providing a reason. Written informed consent was obtained in accordance with the Declaration of Helsinki, and participant confidentiality and anonymity were maintained throughout the study.

### Data analysis

Data were analyzed using IBM SPSS Statistics for Windows, version 22.0 (IBM Corp., Armonk, NY, United States). Descriptive statistics, including frequencies, percentages, means, and standard deviations, were calculated. The normality of continuous variables was assessed using skewness and kurtosis values, which fell within acceptable limits. Accordingly, parametric statistical methods were applied.

A dependent-samples *t*-test was used to compare pre- and postoperative attention control and perceived stress levels. Pearson correlation analysis was conducted to examine the relationships between noise levels, perceived stress, and attention control. A significance level of *P* < 0.05 was accepted for all statistical tests. Analyses were performed only on complete cases; forms containing missing data were excluded from the analysis.

## Results

The demographic and professional characteristics of the 47 operating room nurses who participated in the study are presented in Table 1. Of the participants, 72.3% were female, with a mean age of 31.19 ± 6.88 yr. The majority held an undergraduate degree (66.0%), and the mean professional experience was 9.11 ± 8.02 yr. Nurses worked across a range of surgical specialties, most frequently in orthopedics and traumatology (21.3%) and general surgery (17.0%). Open surgical procedures constituted 74.5% of the observed cases (Table 1).

**Table 1:** Demographic and professional characteristics of operating room nurses.

Characteristics	Frequency (*n*)	Percentage (%)
Gender		
Female	34	72.3
Male	13	27.7
Educational status		
Health vocational high school	3	6.4
Associate degree	8	17.0
Undergraduate degree	31	66.0
Graduate degree	5	10.6
Surgical field of practice		
General surgery	8	17.0
Ear, nose, and throat	6	12.8
Neurosurgery	5	10.6
Ophthalmology	5	10.6
Urology	3	6.4
Cardiovascular surgery	4	8.5
Orthopedics and traumatology	10	21.3
Plastic and reconstructive surgery	6	12.8
Predominant surgical technique		
Open surgery	35	74.5
Laparoscopic surgery	18	25.5

Variables	Mean	SD
Age (yr)	31.19	6.88
Professional experience (yr)	9.11	8.02
Surgical duration (h)^a^	2.27	1.22

SD, standard deviation.

^a^Mean duration of surgical procedures observed during data collection.

A total of 611 noise measurements were recorded across 13 standardized stages of the surgical workflow. The overall mean noise level was 66.57 ± 5.33 dB, with a maximum recorded value of 87 dB. Noise levels varied within a relatively narrow range across workflow stages, as detailed in Table 2. The highest average noise levels were observed during the introduction of additional equipment into the operating room, patient transfer from the stretcher to the operating table, and patient movement out of the room. In contrast, the lowest average noise levels were recorded before patient entry and after the patient had left the operating room. Peak noise exposure values were primarily associated with equipment movement and patient transfer activities (Table 2).

**Table 2:** Instantaneous noise level measurements across workflow stages (dB).

Surgical workflow stage	*N*	Mean ± SD	Min–Max
Before patient entry into the operating room	47	55.06 ± 6.44	38.00 to 70.00
Patient transfer into the operating room	47	65.43 ± 7.93	53.00 to 82.00
Transfer from stretcher to operating table	47	67.96 ± 7.72	52.00 to 81.00
Induction of anesthesia	43^a^	66.58 ± 10.04	13.00 to 79.00
Surgical team positioning around the table	47	67.55 ± 6.51	53.00 to 80.00
Initial surgical incision	47	66.47 ± 6.24	53.00 to 80.00
Entry of an additional team member	47	69.30 ± 6.18	52.00 to 79.00
Opening of the operating room door	47	69.21 ± 6.73	52.00 to 79.00
Entry of additional equipment (eg, portable X-ray)	23^a^	72.61 ± 6.08	54.00 to 83.00
Emergence from anesthesia	36^a^	70.78 ± 6.40	54.00 to 79.00
Transfer from operating table to stretcher	47	70.23 ± 5.61	55.00 to 79.00
Patient exit from the operating room	47	69.77 ± 6.41	52.00 to 87.00
After patient exit	47	60.00 ± 5.77	44.00 to 69.00
Overall mean noise level	47	66.57 ± 5.33	51.80 to 73.46

Min–Max values represent the range of instantaneous point measurements recorded across repeated observations within each workflow stage and do not reflect time-integrated measurements.

SD, standard deviation.

^a^The number of observations varied across workflow stages depending on procedure characteristics and equipment use.

Preoperative and postoperative perceived stress and attention control scores showed no statistically significant changes. The mean perceived stress score increased slightly from 5.36 ± 4.08 before surgery to 5.61 ± 3.66 after surgery (*t*(46) = −0.51, *P* = 0.61). Similarly, attention control scores demonstrated a minimal increase from 58.12 ± 11.39 to 58.25 ± 11.78, which was not statistically significant (*t*(46) = −0.16, *P* = 0.87). Effect sizes for both variables were negligible (Cohen's *d* ≤ 0.07), and confidence intervals did not indicate meaningful change. Although not statistically significant, perceived coping scores approached significance (*P* = 0.06), suggesting a potential trend.

The relationships between mean noise levels and psychological variables are presented in Table 3. Pearson correlation analysis demonstrated weak and statistically nonsignificant associations between mean operating room noise levels and perceived stress both before surgery (*r* = −0.09, *P* = 0.52) and after surgery (*r* = −0.12, *P* = 0.38). Similarly, correlations between mean noise levels and attention control scores were weak and not statistically significant in both the preoperative (*r* = 0.02, *P* = 0.86) and postoperative (*r* = 0.04, *P* = 0.76) periods (Table 3).

**Table 3:** Correlations between mean noise levels and psychological scale scores before and after surgery.

Time point	Scale	*r*	*P*
Pre-procedure	Perceived stress	−0.09	0.52
	Perceived coping	−0.09	0.52
	Perceived Stress Scale (total score)	−0.10	0.49
	Attention Control Scale (total score)	0.02	0.86
Post-procedure	Perceived stress	−0.12	0.38
	Perceived coping	−0.11	0.43
	Perceived Stress Scale (total score)	−0.13	0.35
	Attention Control Scale (total score)	0.04	0.76

*r*, Pearson correlation coefficient; *P*, significance level.

Comparisons of mean noise levels according to surgical field of practice are presented in Table 4. A statistically significant difference was observed between surgical specialties (*F* = 2.81, *P* = 0.01). Post hoc comparisons (Bonferroni-adjusted) indicated that noise levels were significantly lower in cardiovascular surgery compared to ear, nose, and throat (ENT), ophthalmology, general surgery, orthopedics and traumatology, plastic and reconstructive surgery, and urology (*P* < 0.05). In addition, noise levels in ENT and ophthalmology were significantly higher than in neurosurgery (*P* < 0.05). Conversely, no statistically significant difference in mean noise levels was identified between open and laparoscopic surgical techniques (*t* = −0.74, *P* = 0.34) (Table 4).

**Table 4:** Comparison of mean noise levels according to surgical field of practice and surgical technique.

Variable	Category	*N*	Mean ± SD (dB)	Test statistic	*P*
Surgical field of practice	General surgery	8	68.14 ± 4.39	*F* = 2.81	0.01
	Ear, nose, and throat (ENT) surgery	6	69.49 ± 2.41		
	Neurosurgery	5	63.70 ± 7.36		
	Ophthalmology	5	70.17 ± 2.10		
	Urology	3	69.40 ± 3.35		
	Cardiovascular surgery	4	59.04 ± 5.89		
	Orthopedics and traumatology	10	66.03 ± 4.29		
	Plastic and reconstructive surgery	6	65.43 ± 5.95		
Surgical technique	Open surgery	35	66.23 ± 5.87	*t* = −0.74	0.34
	Laparoscopic surgery	12	67.56 ± 3.30		

Post hoc comparisons indicate statistically significant differences between the specified surgical fields.

SD, standard deviation; *F*, 1-way analysis of variance; *t*, independent-samples *t*-test.

Overall, the findings indicate that noise levels showed moderate variation across workflow stages and remained within a moderate range, with increases observed during high-activity phases such as equipment movement and patient transfer. However, these differences should be interpreted with consideration of the measurement accuracy (±2 dB) of the sound level meter. Despite these measured noise levels, no statistically significant short-term changes were observed in perceived stress or attention control measures.

## Discussion

This study demonstrates that noise levels in operating rooms are not constant across the surgical process but show moderate variation between workflow stages. Although the overall mean noise level was 66.57 dB, short-duration peak levels reached up to 87 dB, particularly during equipment movement and patient transfer. However, these peaks, while approaching levels reported in occupational exposure guidelines, do not represent time-weighted average exposure and should therefore be interpreted cautiously. Importantly, the observed noise levels remained within a moderate range and below commonly cited occupational hearing damage thresholds (eg, 85 dBA). However, evidence suggests that noise levels in the range of 60 to 70 dBA may still interfere with communication, concentration, and cognitive performance in complex clinical environments (Wahr and Abernathy 2024; Liu et al. 2025). In this context, the measured noise levels may have functional significance, particularly during workflow phases requiring clear communication and sustained attention (Hughes et al. 2025). Similar stage-dependent elevations have been reported in previous operating room noise studies, especially during high-activity and transition phases (Arabacı and Önler 2021; McLeod et al. 2021). These findings support the concern that reliance on time-averaged measurements alone may limit the identification of workflow-dependent variations in environmental noise conditions. These observations should be interpreted in light of the point-based measurement approach used in this study. Future studies incorporating personal noise dosimetry may provide time-integrated exposure data and allow more precise evaluation of noise variations across surgical workflow phases.

Noise levels differed across surgical specialties, consistent with earlier reports that workflow structure, team activity patterns, and equipment intensity shape the acoustic profile of operating rooms (Giv et al. 2017; Keller et al. 2018). Specialties characterized by frequent equipment repositioning and staff movement showed higher exposure levels. In contrast, no significant differences were observed between open and laparoscopic approaches, suggesting that exposure variability is driven more by workflow dynamics than by surgical technique alone. This reinforces the need for exposure assessment models that incorporate task and workflow characteristics rather than procedure labels only.

No significant short-term changes were observed in perceived stress or attention control among surgical nurses despite the presence of peak noise exposures (Wang et al. 2025). Comparable findings have been described in studies involving experienced operating room personnel (Padmakumar et al. 2017; Gülşen et al. 2021). Adaptation, professional experience, and routine exposure to demanding environments may prevent immediate adverse stress and attention control responses. In addition, the study design, which focused only on the first surgical procedure of the day, may have limited the detection of potential changes associated with cumulative workload and prolonged exposure to noise throughout the workday. Therefore, the absence of short-term changes in self-reported indicators should not be interpreted as evidence of negligible exposure impact.

The weak associations between average noise levels and psychological measures further suggest that conventional self-report tools and short observation windows may have limited sensitivity for detecting the effects of brief, peak noise events. Functionally important effects of noise are likely to occur during specific communication-critical moments rather than as generalized short-term psychological shifts (Li et al. 2025). Repeated exposure to such peak noise conditions may still contribute to cumulative cognitive load, communication degradation, and increased error vulnerability over time, even when immediate scale-based measures remain unchanged (McLeod et al. 2021; Nasri et al. 2023; Louis et al. 2024).

Taken together, the findings support viewing operating room noise as a dynamic occupational exposure characterized by task-dependent peaks rather than as a uniform background condition. Workflow transitions—including patient transfer, equipment entry, and team reorganization—emerged as periods with relatively higher noise levels within the observed range, which coincide with elevated coordination demands and reduced tolerance for error. Although international bodies such as the World Health Organization and the National Institute for Occupational Safety and Health recognize the importance of peak noise exposure, current guidance offers limited methodological direction for incorporating task variability into routine exposure assessment practice (World Health Organization 1999; National Institute for Occupational Safety and Health 2018; World Health Organization 2024). This gap may lead to incomplete occupational risk characterization in complex clinical workplaces.

From an occupational health practice perspective, workflow-aligned noise monitoring may improve hazard identification and prioritization. Because surgical nurses remain present across all perioperative stages, they experience repeated exposure to transition-related peaks. Risk management strategies based only on overall exposure averages may therefore overlook the moments of highest functional vulnerability. Task-resolved exposure assessment can provide a more precise basis for targeted preventive measures.

Practical risk control approaches proposed in prior studies include regulating non-essential movement during critical phases, optimizing equipment positioning, reducing avoidable door openings, and standardizing communication protocols (Kol et al. 2015; Grant et al. 2021). Structured verbal communication and quiet-phase practices may help maintain message clarity during high-noise intervals. These measures can be classified primarily as administrative controls within the occupational health and safety hierarchy, as they aim to modify work practices rather than eliminate the noise source. While engineering controls—such as the use of noise-dampening materials, equipment redesign, or alarm volume optimization—are generally preferred for hazard reduction, their implementation may be limited in complex surgical environments where workflow flexibility and sterility requirements must be maintained. In this context, administrative and behavioral strategies may provide practical and immediately applicable approaches to reducing the functional impact of noise, particularly during communication-critical phases of surgical care.

Overall, the results indicate that operating room noise should be considered a workflow-dependent occupational hazard rather than solely an environmental comfort factor. Incorporating task-based exposure characterization into occupational noise risk assessment may improve the accuracy of hazard detection and better align control strategies with real-world clinical workflow.

Several limitations should be considered. The study was conducted in a single center, which may limit external generalizability. Measurements were based on repeated point readings rather than continuous dosimetry, and individual worker movement and task distribution were not separately modeled. In addition, measurements were obtained at a fixed position approximately 1.5 m above the floor and do not necessarily represent the individual hearing zone of each nurse. Therefore, the findings should be interpreted as environmental noise measurements rather than precise estimates of personal exposure. The study did not stratify participants according to professional experience, which may influence individual responses to noise exposure. Experienced nurses may demonstrate adaptive coping mechanisms, whereas less experienced staff may be more vulnerable to distraction and cognitive load under similar environmental conditions. In addition, measurements were limited to the first surgical procedure of the day, which may not reflect variations in noise exposure associated with cumulative workload, staff fatigue, or increased activity levels later in the workday. As a result, the findings may underestimate peak variability under more demanding operating conditions. Psychological outcomes were evaluated over a short time frame and may not capture cumulative exposure effects. Moreover, the use of self-reported measures may have limited sensitivity for detecting subtle or transient changes in stress and attention control. Future studies should incorporate personal noise dosimetry to capture time-integrated exposure and allow more accurate assessment of individual noise exposure across different surgical workflow phases. Nevertheless, the structured workflow-stage measurement framework and real-time field data provide useful evidence for task-based occupational noise exposure assessment in operating room settings.

## Conclusion

This field measurement study demonstrated that noise levels in operating rooms show workflow-dependent variation rather than marked differences across the surgical process and can reach short-duration peak levels during safety-critical phases such as equipment movement and patient transfer. Although average noise levels remained below commonly cited occupational hearing loss prevention thresholds (eg, 85 dBA for time-weighted average exposure), the presence of sudden, workflow-dependent peaks indicates that reliance on mean noise level measurements alone may lead to underestimation in environmental noise variability.

The absence of significant short-term changes in perceived stress and attention control should be interpreted cautiously. These findings do not suggest that noise exposure is harmless, but rather that commonly used psychological indicators may have limited sensitivity for detecting the acute or cumulative effects of brief, peak noise events. Repeated peak exposures may still adversely affect communication quality, coordination reliability, and patient safety over time, particularly as noise levels in the range of 60 to 70 dBA have been associated with interference in communication and cognitive task performance in complex clinical settings (Peisl et al. 2024).

From an occupational health and exposure assessment perspective, the findings support moving beyond sole reliance on average noise metrics toward task-based and workflow-aligned environmental noise evaluation. Integrating stage-based noise monitoring into routine workplace exposure and risk assessment practices may enable more accurate hazard identification and more targeted risk control strategies. Practical controls—including managing staff movement during critical phases, optimizing equipment placement, reducing avoidable door openings, and standardizing communication protocols—represent primarily administrative control measures, which may offer feasible and immediately applicable approaches in surgical environments where engineering controls are more difficult to implement.

Overall, operating room noise should be recognized as a dynamic, workflow-dependent environmental factor rather than solely an environmental comfort issue. Task-resolved environmental noise assessment approaches, although based on point measurements in the present study, can support improved understanding of workflow-related variations and inform more context-sensitive strategies for enhancing communication, coordination, and patient safety in surgical settings.

## Data Availability

The datasets generated and/or analyzed during the current study are not publicly available due to institutional data protection policies but are available from the corresponding author on reasonable request.
